# Association between the circulating very long-chain saturated fatty acid and cognitive function in older adults: findings from the NHANES

**DOI:** 10.1186/s12889-024-18478-x

**Published:** 2024-04-16

**Authors:** Yanxin Shen, Chunxiao Wei, Yezi Taishi, Guimei Zhang, Zhan Su, Panpan Zhao, Yongchun Wang, Mingxi Li, Yingshi Ji, Li Sun

**Affiliations:** 1 Department of Neurology and Neuroscience Center, The First Hospital of Jilin University, Xinmin Street 1#, 130021 Changchun, China; 2 Cognitive Center, Department of Neurology, The First Hospital of Jilin University, Changchun, China; 3 Department of Cadre Ward, The First Hospital of Jilin University, Changchun, China; 4 Department of Pharmacology, Physiology and Cell Biology, College of Basic Medical Sciences, Jilin University, Changchun, China

**Keywords:** Cognitive function, Fatty acid, Very long-chain saturated fatty acids, NHANES

## Abstract

**Background:**

Age-related cognitive decline has a significant impact on the health and longevity of older adults. Circulating very long-chain saturated fatty acids (VLSFAs) may actively contribute to the improvement of cognitive function. The objective of this study was to investigate the associations between arachidic acid (20:0), docosanoic acid (22:0), tricosanoic acid (23:0), and lignoceric acid (24:0) with cognitive function in older adults.

**Methods:**

This study used a dataset derived from the 2011–2014 National Health and Nutrition Examination Survey (NHANES). A total of 806 adults (≥ 60 years) were included who underwent comprehensive cognitive testing and plasma fatty acid measurements. Multivariable linear regression, restricted cubic spline (RCS), and interaction analyses were used to assess associations between VLSFAs and cognitive function. Partial Spearman’ s correlation analysis was used to examine the correlations between VLSFAs and palmitic acid (16:0), high-density lipoprotein cholesterol, low-density lipoprotein cholesterol, total cholesterol, triglycerides, systemic inflammatory markers, and dietary nutrients.

**Results:**

Multivariable linear regression analysis, adjusting for sociodemographic, clinical conditions, and lifestyle factors, showed that 22:0 and 24:0 levels were positively associated with better global cognitive function (β = 0.37, 95% confidence interval [CI] = 0.01, 0.73; β = 0.73, 95% CI = 0.29, 1.2, respectively) as well as better CEARD-DR Z-score (β = 0.82, 95% CI = 0.36, 1.3 and β = 1.2, 95% CI = 0.63, 1.8, respectively). RCS analysis showed linear associations between higher 22:0 and 24:0 levels and better cognitive performance in both global cognitive function and CERAD-DR tests.

**Conclusions:**

The study suggests that higher levels of 22:0 and 24:0 are associated with better global cognitive function in older adults. 22:0 and 24:0 may be important biomarkers for recognizing cognitive impairment, and supplementation with specific VLSFAs (22:0 and 24:0) may be an important intervention to improve cognitive function. Further studies are needed to elucidate the underlying biological mechanisms between VLSFAs and cognitive function.

**Supplementary Information:**

The online version contains supplementary material available at 10.1186/s12889-024-18478-x.

## Introduction

As life expectancy continues to increase, coupled with global population growth, it is estimated that the global incidence of dementia will increase from 57.4 million cases in 2019 to 152.8 million cases in 2050 [1]. Dementia is a complex disease involving multimolecular, multisystem, and network interactions. Cognitive function decline is a multifactorial process, and epidemiological studies have revealed that genetics, environmental factors, lifestyle, and health conditions (e.g., obesity, hypertension, and diabetes mellitus) can individually or synergistically mediate pathological processes (e.g., inflammation, oxidative stress, and neuronal synaptic dysfunction) [2–6]. In particular, age is an independent risk factor for dementia, with the risk of developing dementia increasing with age. Age-related cognitive decline is a significant health challenge for older individuals and is characterized by impairments in conceptual reasoning, memory, executive function, sustained attention, and processing speed [7]. Cognitive function decline and dementia place a heavy economic burden on society. To address the challenges of global aging, the identification of new factors to prevent cognitive decline is critical for public health. Fatty acids, as integrated markers of diet and metabolism, have a potential impact on cognitive function. Omega-3 long-chain polyunsaturated fatty acids (PUFAs), such as docosahexaenoic and eicosapentaenoic acid, have shown promising results in preventing cognitive decline and reducing the risk of dementia [8–10]. Recent research using metabolomics data from the UK Biobank has shown inverse associations between plasma levels of PUFAs and the risk of dementia [11, 12]. However, there is limited evidence on the effects of very long-chain saturated fatty acids (VLSFAs) on cognitive function.

Plasma VLSFAs, which are saturated fatty acids with ≥ 20 carbon atoms, have recently received considerable attention for their beneficial effects on health [13]. Circulating VLSFAs are derived from diet and endogenous metabolism. Some nuts and seeds, including peanuts, macadamia nuts, and canola oils, contain the most VLSFAs [13]. According to previous studies, high levels of arachidic acid (20:0), docosanoic acid (22:0), and lignoceric acid (24:0) are associated with a lower risk of diabetes mellitus [14], and various cardiovascular disease, including atrial fibrillation [15], heart failure [16], and coronary heart disease (CHD) [17]. Given the findings of age-related degenerative diseases, elevated levels of VLSFAs may be beneficial in improving cognitive function. Fatty acids are involved in several biological processes in the brain, and their effects on inflammation vary depending on their chain length. The Insulin Resistance Atherosclerosis Study found that, among even-chain saturated fatty acids, those with shorter chain lengths (myristic acid [14:0] and palmitic acid [16:0]) exhibited positive correlations with pro-inflammatory markers and negative correlations with adiponectin, whereas even-chain VLSFAs with longer chain lengths (20:0 and 22:0) and odd-chain SFA (15:0) displayed inverse correlations [18]. However, it remains unclear whether VLSFAs improve cognitive function through anti-inflammatory effects.

To our knowledge, few studies have investigated the relationship between plasma VLSFAs and cognitive function. Therefore, we used data from the National Health and Nutrition Examination Survey (NHANES) to specifically examine the associations between plasma 20:0, 22:0, tricosanoic acid (23:0), 24:0, and total VLSFAs with cognitive performance.

## Methods

### Study design and population

The NHANES is intended to evaluate the health and dietary conditions of individuals in the United States and is representative of the entire nation. This study was sanctioned and supported by the U.S. Centers for Disease Control and Prevention and evaluated and endorsed by the National Center for Health Statistics Research Ethics Review Board. Data were collected every two years using a complex multistage probability sampling design. All participants provided informed consent.

We followed the Strengthening the Reporting of Observational Studies in Epidemiology (STROBE) guidelines for reporting on cross-sectional studies and used two data cycles (2011–2012, 2013–2014) obtained from the NHANES. The study included 3,632 participants aged ≥ 60 years who completed four cognitive function tests; those with incomplete data on plasma fatty acids were excluded (*n* = 2,128), resulting in the inclusion of 806 participants in the study (Supplementary Fig. 1).

### Plasma fatty acids, lipids, and systemic inflammatory variables

Detailed instructions for sample collection and processing are available at https://wwwn.cdc.gov/nchs/data/nhanes/2015-2016/manuals/2016_MEC_Laboratory_Procedures_Manual.pdf. Electron capture negative-ion mass spectrometry was used to examine fatty acids, using a technique based on a modification of the approach established by Lagerstedt et al. [19]. Plasma fatty acid levels were measured using this method. Fatty acid units were converted from µmol/L to percentage of total fatty acids. Total VLSFAs is the sum of 20:0, 22:0, 23:0, and 24:0 as a percentage of total fatty acids. A Modular P Chemistry Analyzer (Roche, Basel, Switzerland) was used to measure the concentrations of triglycerides, high-density lipoprotein (HDL) cholesterol, and total cholesterol after enzymatic treatment of the samples. Total cholesterol, triglycerides, and HDL cholesterol were used to calculate low-density lipoprotein (LDL) cholesterol levels according to the Friedewald calculation: LDL cholesterol = (total cholesterol) - (HDL cholesterol) - triglycerides/5. A DxH 800 instrument (Beckman Coulter, Brea, CA) was used at the NHANES Mobility Examination Center (MEC) to perform a comprehensive analysis of the blood samples, including measurements of white blood cell (WBC), neutrophil, lymphocyte, monocyte, and platelet count. The DxC800 used a bichromatic digital endpoint method to determine albumin concentration. The neutrophil-to-lymphocyte ratio (NLR) and the neutrophil-to-albumin ratio (NAR) were also calculated. The systemic immune-inflammation index (SII) and systemic inflammatory response index (SIRI) were calculated according to the following formulas: SII = (platelet count × neutrophil count)/lymphocyte count; SIRI = (neutrophil count × monocyte count)/lymphocyte count.

### Cognitive function variables

At the MEC, trained interviewers assessed participants’ cognitive function after an in-person private interview. Prior to commencing the assessment, the interviewer requested participants to grant consent for recording, primarily for marking and quality control purposes. The Consortium to Establish a Registry for Alzheimer’s Disease (CERAD) Word Learning, the Animal Fluency Test (AFT), and the Digit Symbol Substitution Test (DSST) were administered. The CERAD Word Learning subtest evaluates the capacity for immediate recall (CERAD-IR) and delayed recall (CERAD-DR) of fresh verbal information (memory subdomain), consisting of three consecutive learning trials and one delayed recall trial, with scores ranging from 0 to 10 for each trial. The total score for three consecutive learning trials ranged from 0 to 30. After administration of the AFT and DSST, the CERAD-DR test was conducted. The AFT evaluates categorical verbal fluency, a part of executive function, on a scale of 3–39. The DSST, a component of the Wechsler Adult Intelligence Scale (WAIS III), evaluates the participants’ cognitive abilities in terms of processing speed, sustained attention, and working memory, with scores ranging from 0 to 105. The composite Z-score was calculated by averaging the Z-scores of the four cognitive tests (CERAD-IR, CERAD-DR, AFT, and DSST). The score was calculated as Z = (x-m)/σ, where x represents the raw score, m represents the mean score, and σ represents the standard deviation (SD).

### Covariates

Sociodemographic covariates included age, sex, race (non-Hispanic White, non-Hispanic Black, other Hispanic, Mexican American, other/multiracial), education (less than 9th grade, 9–11th grade, high school graduate/GED, some college or associate’s degree, college graduate or above), and family income-to-poverty ratio (PIR). Lifestyle covariates included smoking status (current, former, or never smoker), alcohol intake (≥ 12 drinks/year), body mass index (BMI), and waist circumference. Standardized questionnaires were used to assess smoking status and alcohol intake. Trained health technicians at the MEC assessed participants’ BMI and waist circumference. Health-related covariates included hypertension, diabetes mellitus, stroke, and CHD. The survey questions asked whether individuals had been told by a physician or other health care professional that they had hypertension, diabetes mellitus, stroke, or CHD. A “yes” response to this question was coded as hypertension, diabetes mellitus, stroke, or CHD. The Patient Health Questionnaire (PHQ-9) is a 9-item screening tool that asks patients how often they have experienced depressive symptoms in the past 2 weeks to assess depression, with a total score of 0–27 [20].

### Statistical analysis

We employed the weighting method recommended by the NHANES Reporting Guidelines for analysis of complex data by dividing the 2-year FAS weights by 2 to determine the 4-year sample weights. Continuous variables were presented as the weighted means ± SD. Categorical variables were expressed as the numbers (n) and percentages (%). The Rau–Scott chi-squared test was used for categorical variables, and the rank-sum test was used for continuous variables. Plasma VLSFAs levels were categorized into four levels (quartiles 1–4, Q1–Q4) based on their distribution, and Q1, which had the lowest level, was used as the reference group. Partial Spearman’s correlation coefficients (rs) were used to evaluate the correlations between the four VLSFAs and between VLSFAs and 16:0, plasma lipids (HDL cholesterol, LDL cholesterol, total cholesterol, triglycerides), systemic inflammatory markers (WBC, neutrophil, lymphocyte count, NLR, NAR, SII, and SIRI), and dietary nutrient intake (total energy, protein, carbohydrate, fiber, sugar, total fat, total monounsaturated fat, total polyunsaturated fat, and total cholesterol).

Multivariable linear regressions were developed to investigate the associations between VLSFAs and the composite Z-score, CERAD-IR Z-score, CERAD-DR Z-score, AFT Z-score, and DSST Z-score. Model 1 was unadjusted. Model 2 was adjusted for age, sex, race, education, PIR. Model 3 was adjusted for age, sex, race, education, PIR, BMI, waist circumference, alcohol intake, smoking status, hypertension, diabetes mellitus, stroke, CHD, and depression score. These confounders have a significant impact on cognitive function [21–23]. Combined with previous relevant studies, we selected these factors for adjustment [24, 25]. In addition, lipid metabolism plays an important role in cognitive impairment [26–28]. To clarify whether these factors affect the relationships between VLSFAs and cognitive function, we further adjusted for 16:0, HDL cholesterol, and triglycerides. Model 4 was adjusted for 16:0 based on Model 3. Model 5 was adjusted for HDL cholesterol based on Model 3. Model 6 was adjusted for triglycerides based on Model 3. Model 7 was adjusted for 16:0, triglycerides, and HDL cholesterol based on Model 3. Results are presented as estimated effect size (β) and 95% confidence interval (CI). To assess the potential relationship (either linear or nonlinear) between plasma VLSFAs levels and participants’ cognitive test scores, a 4-knot restricted cubic spline (RCS) analysis was used. Levels of 22:0, 23:0, 24:0 and total VLSFAs were used as limiting variables. We further examined potential interactions between VLSFAs and sex, race, education, smoking status, alcohol intake, diabetes mellitus, hypertension, stroke, and CHD in relation to associations of the composite Z-scores. R software (version 4.2.1; R Foundation, Vienna, Austria) was used for statistical analyses. The significance level for this study was defined as a two-tailed *P* value < 0.05.

## Results

### Characteristics of the study population

The participant mean age (SD) was 69 (6) years, and 43% were male (Table 1). The mean (SD) level of each VLSFA was 0.51% (0.13) for 24:0, 0.26% (0.07) for 23:0, 0.6% (0.16) for 22:0, and 0.21% (0.05) for 20:0 (Supplementary Table 1). The characteristics of the 806 participants are presented in Table 1, along with the distribution of these characteristics across quartiles of 24:0. Participants in the highest quartile of 24:0 were more likely to be female and non-Hispanic Black. They were also more likely to have higher education, cognitive test scores, total dietary energy intake, and HDL and LDL cholesterol. Additionally, they were less likely to have diabetes mellitus and hypertension and more likely to have lower depression scores, BMI, waist circumference, triglycerides, WBC, neutrophil count, NAR, and SIRI.

**Table 1:** Characteristics of the study population and stratified by lignoceric acid (24:0)

Characteristics	Overall	Quartile of lignoceric acid (24:0)				*P* value
		Q1 ( < = 0.41)	Q2 (0.41–0.50)	Q3 (0.50–0.60)	Q4 (> 0.60)	
**Number**	**806**	**211**	**220**	**199**	**176**	
**Weighted number**	**33,459,737**	**8,367,754**	**8,434,947**	**8,305,116**	**8,351,919**	
**Continuous variables**, mean ± SD						
Age (year)	69 ± 6	71 ± 6	69 ± 7	69 ± 6	68 ± 6	< 0.001 ^***^
PIR	3.13 ± 1.58	2.89 ± 1.51	3.03 ± 1.56	3.15 ± 1.67	3.45 ± 1.55	0.004 ^**^
BMI, kg/m^2^	29 ± 6	31 ± 6	29 ± 6	28 ± 6	27 ± 6	< 0.001 ^***^
Waist circumference (cm)	102 ± 15	108 ± 15	102 ± 13	101 ± 15	98 ± 15	< 0.001 ^***^
Composite Z-score	0.25 ± 0.7	0.02 ± 0.75	0.20 ± 0.72	0.19 ± 0.79	0.58 ± 0.73	< 0.001 ^***^
CERAD-IR Z-score	0.21 ± 0.94	-0.01 ± 1.01	0.19 ± 0.85	0.11 ± 0.93	0.58 ± 0.85	< 0.001 ^***^
CERAD-DR Z-score	0.18 ± 0.97	-0.05 ± 0.97	0.09 ± 0.94	0.13 ± 0.99	0.55 ± 0.89	< 0.001 ^***^
AFT Z-score	0.25 ± 1.01	0.07 ± 0.95	0.27 ± 0.99	0.16 ± 1.01	0.50 ± 1.05	0.018 ^*^
DSST Z-score	0.34 ± 0.98	0.07 ± 0.93	0.27 ± 0.94	0.35 ± 0.98	0.68 ± 0.99	< 0.001 ^***^
Depression score	3.1 ± 4.4	4.3 ± 5.9	3.1 ± 3.7	2.7 ± 3.7	2.5 ± 3.8	< 0.001 ^***^
Total energy intake, kcal	1,866 ± 683	1,749 ± 664	1,872 ± 647	1,925 ± 769	1,918 ± 635	0.010 ^*^
Triglycerides, mg/dL	121 ± 66	189 ± 78	123 ± 42	100 ± 41	72 ± 26	< 0.001 ^***^
Total cholesterol, mg/dL	192 ± 42	185 ± 42	191 ± 42	190 ± 45	201 ± 39	0.14
HDL cholesterol, mg/dL	57 ± 17	47 ± 12	55 ± 14	58 ± 15	69 ± 18	< 0.001 ^***^
LDL cholesterol, mg/dL	110 ± 36	101 ± 36	111 ± 36	112 ± 39	117 ± 32	0.021 ^*^
WBC, ×10^3^ cells/µL	6.67 ± 2.02	7.19 ± 1.78	6.68 ± 2.00	6.45 ± 1.78	6.35 ± 2.35	< 0.001 ^***^
Lymphocyte, ×10^3^ cells/µL	1.83 ± 0.75	1.95 ± 0.68	1.81 ± 0.67	1.74 ± 0.55	1.83 ± 1.00	0.080
Neutrophils, ×10^3^ cells/µL	4.01 ± 1.59	4.39 ± 1.46	4.07 ± 1.56	3.86 ± 1.39	3.73 ± 1.85	< 0.001 ^***^
NLR	2.47 ± 1.58	2.58 ± 1.52	2.46 ± 1.26	2.45 ± 1.27	2.37 ± 2.13	0.3
NAR	0.96 ± 0.39	1.04 ± 0.36	0.98 ± 0.39	0.92 ± 0.32	0.90 ± 0.47	< 0.001 ^***^
SII	553 ± 405	549 ± 329	561 ± 331	561 ± 434	542 ± 502	0.5
SIRI	1.42 ± 1.26	1.57 ± 1.11	1.39 ± 1.05	1.38 ± 0.93	1.35 ± 1.78	0.008 ^**^
**Categorical variables**, n (%)						
Male	373 (43)	104 (46)	110 (44)	86 (47)	73 (37)	0.5
Race						< 0.001 ^***^
Non-Hispanic White	426 (81)	115 (80)	115 (80)	100 (79)	96 (84)	
Non-Hispanic Black	159 (8.5)	16 (3.2)	37 (8.3)	47 (10)	59 (13)	
Other Hispanic	94 (4.0)	33 (6.1)	32 (5.4)	20 (3.2)	9 (1.3)	
Other/multiracial	58 (3.3)	21 (4.6)	15 (3.0)	15 (3.8)	7 (1.6)	
Mexican American	69 (3.5)	26 (5.7)	21 (3.6)	17 (3.6)	5 (1.0)	
Education						0.034 ^*^
Less than 9th grade	90 (5.7)	35 (9.0)	32 (7.4)	18 (4.7)	5 (1.9)	
9-11th grade	119 (12)	36 (12)	30 (11)	33 (16)	20 (7.8)	
High school graduate/GED	190 (22)	47 (22)	55 (24)	43 (26)	44 (18)	
Some college or associate’s degree	216 (30)	56 (33)	54 (30)	52 (24)	54 (33)	
College graduate or above	191 (30)	37 (24)	49 (28)	53 (30)	53 (40)	
Alcohol intake (≥ 12 drinks/year)	550 (73)	136 (70)	146 (70)	143 (76)	126 (77)	0.3
Smoking status						0.8
Current smoker	97 (11)	29 (10)	23 (10)	23 (13)	22 (9.1)	
Former smoker	311 (39)	86 (43)	88 (42)	74 (35)	65 (37)	
Never smoker	398 (50)	96 (47)	109 (48)	102 (52)	89 (54)	
Hypertension	517 (61)	148 (74)	146 (63)	123 (59)	101 (49)	0.005 ^**^
Diabetes mellitus						0.002 ^**^
No	608 (77)	146 (66)	155 (74)	161 (79)	146 (89)	
Borderline	39 (5.4)	7 (3.5)	13 (7.4)	11 (5.8)	8 (5.0)	
Yes	159 (17)	58 (30)	52 (19)	27 (15)	22 (5.8)	
Stroke	55 (6.0)	16 (7.2)	14 (4.5)	15 (7.8)	10 (4.6)	0.5
CHD	72 (9.8)	29 (15)	18 (9.5)	15 (7.9)	10 (6.4)	0.074

^*^P < 0.05, ^**^P < 0.01, ^***^P < 0.001. AFT, Animal Fluency Test; BMI, body mass index; CERAD, Consortium to Establish a Registry for Alzheimer’s Disease; CERAD-IR, CERAD immediate recall; CERAD-DR, CERAD delayed recall; CHD, coronary heart disease; DSST, Digit Symbol Substitution Test; HDL, high-density lipoprotein; LDL, low-density lipoprotein; NLR, neutrophil-to-lymphocyte ratio; NAR, neutrophil-to-albumin ratio; PIR, family income-to-poverty ratio; SII, systemic immune-inflammation index; SIRI, systemic inflammatory response index; SD, standard deviation; WBC, white blood cell

### Associations between plasma VLSFAs and cognitive function

**Fig. 1:**
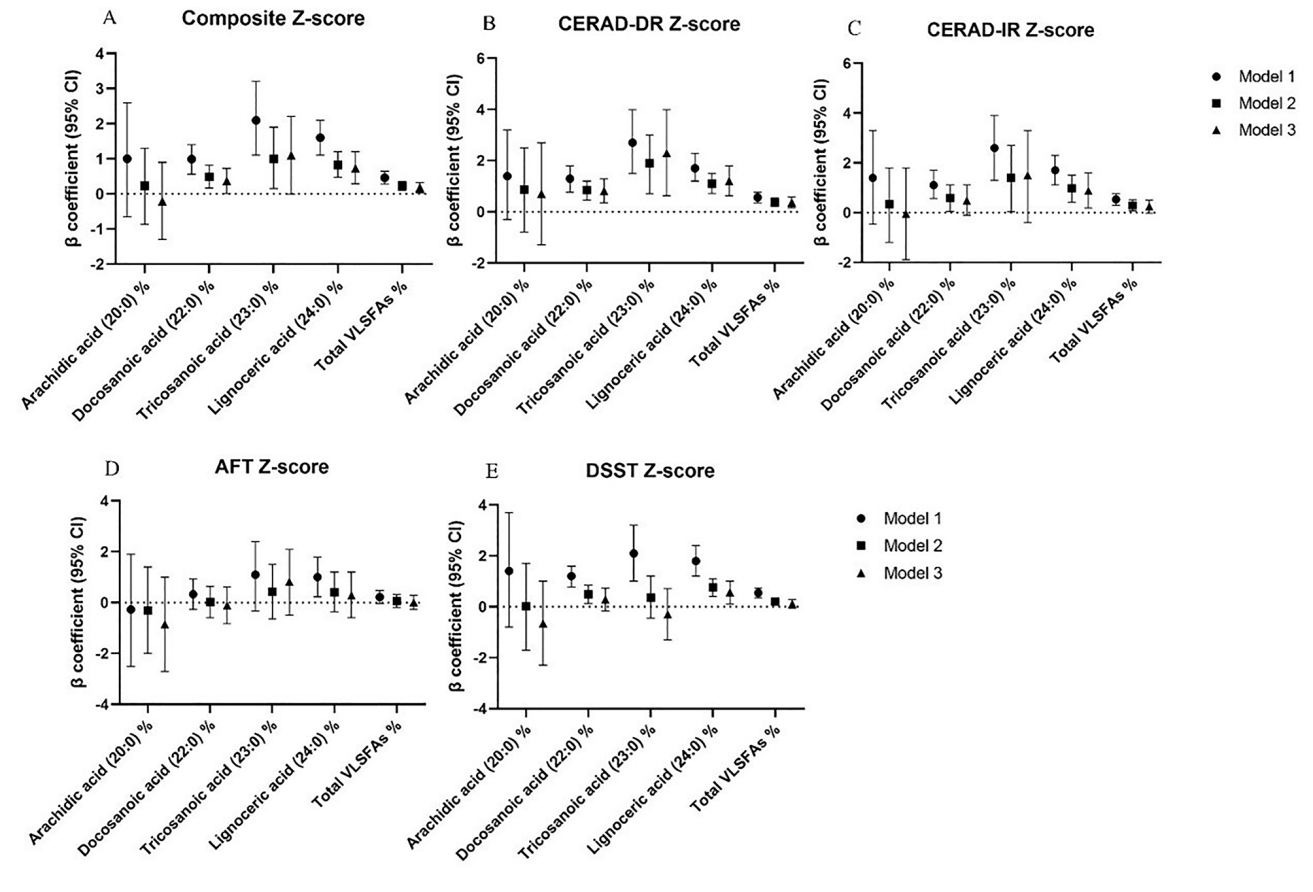
Linear regression models for associations between very long-chain saturated fatty acids (VLSFAs) and four tests of cognitive function (*n* = 806). AFT, Animal Fluency Test; CERAD, Consortium to Establish a Registry for Alzheimer’s Disease; CERAD-IR, CERAD immediate recall; CERAD-DR, CERAD delayed recall; DSST, Digit Symbol Substitution Test; VLSFAs, very long-chain saturated fatty acids. Model 1, did not adjust for any confounders. Model 2, adjusted for age, sex, race, education, and PIR. Model3, adjusted for age, sex, race, education, PIR, BMI, waist circumference, alcohol intake, smoking status, hypertension, diabetes mellitus, stroke and coronary heart disease, depression score

Figure 1. displays the relationships between VLSFAs as continuous variables and cognitive performance. Three models were used in this study. For the global cognitive function, the multivariable linear regression analysis, adjusting for age, sex, race, education, PIR, BMI, waist circumference, alcohol intake, smoking status, hypertension, diabetes mellitus, stroke, CHD, and depression score, showed that the levels of 22:0, 24:0, and total VLSFAs were positively associated with composite Z-scores (β = 0.37, 95% CI = 0.01, 0.73; β = 0.73, 95% CI = 0.29, 1.2; β = 0.18, 95% CI = 0.03, 0.33, respectively). The levels of 22:0, 24:0, and total VLSFAs were positively associated with CEARD DR-Z score (β = 0.82, 95% CI = 0.36, 1.3; β = 1.2, 95% CI = 0.63, 1.8; β = 0.37, 95% CI = 0.16, 0.59, respectively). The associations between 22:0, 24:0 and AFT were not statistically significant in the multivariable linear regression analysis. Furthermore, the levels of 24:0 demonstrated positive associations with CEARD IR-Z score (β = 0.88, 95% CI = 0.19, 1.6) and DSST-Z score (β = 0.57, 95% CI = 0.10, 1.0).

**Fig. 2:**
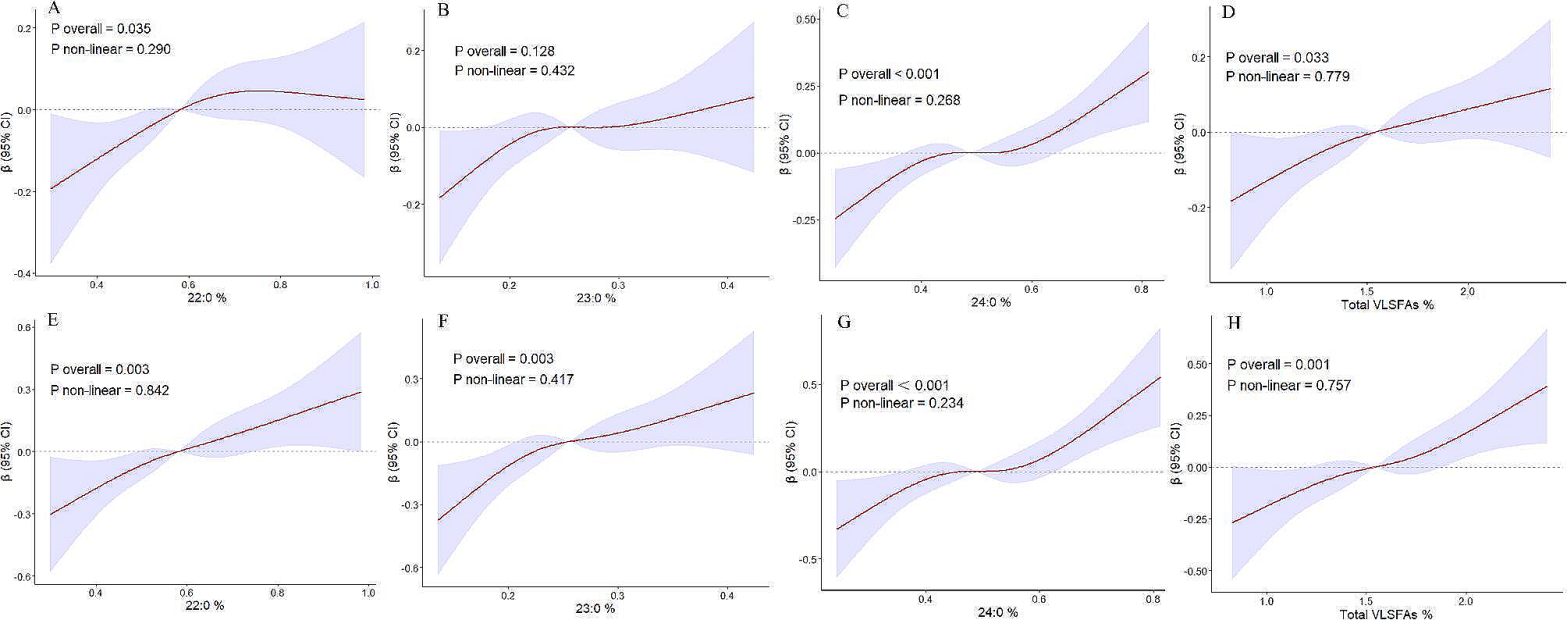
Restricted cubic spline (RCS) analysis between very long-chain saturated fatty acids (VLSFAs) and composite Z-score (**A**-**D**) and CERAD-DR Z-score (**E**-**H)**. CERAD-DR, Consortium to Establish a Registry for Alzheimer’s Disease Word Learning delayed recall

We further analyzed the relationships between VLSFAs as categorical variables and cognitive function. Multivariable linear regression analysis showed that the highest quartiles of 22:0 and 24:0 were both associated with higher composite Z-scores compared to the lowest quartiles (β = 0.22, 95% CI = 0.00, 0.44, *P* trend = 0.045 and β = 0.22, 95% CI = 0.03, 0.41, *P* trend = 0.025, respectively) after adjustment for age, sex, race, education, PIR, BMI, waist circumference, smoking status, alcohol intake, diabetes mellitus, hypertension, stroke, CHD, and depression scores (Model 3), (Supplementary Tables 2 and 3). Further analysis of the relationship between VLSFAs with different cognitive domains revealed that the highest quartiles of 22:0, 24:0, and total VLSFAs were all associated with higher CERAD DR-Z scores compared to the lowest quartiles (β = 0.37, 95% CI = 0.07, 0.67, *P* trend = 0.007; β = 0.36, 95% CI = 0.14, 0.58, *P* trend = 0.005; β = 0.43, 95% CI = 0.12, 0.73, *P* trend = 0.004, respectively) (Supplementary Tables 2, 3 and 4). The highest quartile of 24:0 was associated with higher CERAD IR-Z scores compared to the lowest quartile (β = 0.28, 95% CI = 0.01, 0.55, *P* trend = 0.065) (Supplementary Table 3). However, plasma 24:0, 23:0, 22:0, 20:0, and total VLSFAs levels were not significantly associated with AFT or DSST (Supplementary Tables 2–6). The RCS analysis indicated that the positive associations of higher 22:0 and 24:0 levels with better composite Z-scores and CERAD-DR Z-scores were linear, and a nonlinear test comparing the cubic spline curves with the linear model showed *P* non-line > 0.05 after adjustment for age, sex, race, education, PIR, BMI, waist circumference, alcohol intake, smoking status, hypertension, diabetes mellitus, stroke, CHD, and depression score (Fig. 2).

### Partial Spearman’s correlation analysis between plasma VLSFAs and lipid markers

In partial Spearman’s correlation analysis adjusting for age, sex, race, education, PIR, BMI, waist circumference, alcohol intake, and smoking status, VLSFAs exhibited strong intercorrelations with each other, particularly between 22:0 and 24:0 (rs = 0.94, *P* < 0.001). 20:0, 22:0, 23:0, and 24:0 were negatively correlated with plasma 16:0 (rs = -0.34, -0.43, -0.36, and -0.4, *P* < 0.001). 20:0, 22:0, 23:0, and 24:0 were positively correlated with HDL cholesterol (rs = 0.32, 0.34, 0.35, and 0.4, *P* < 0.001) and negatively correlated with triglycerides (rs = -0.56, -0.62, -0.64, and -0.66, *P* < 0.001). However, VLSFAs showed weaker correlations with LDL and total cholesterol (Table 2).

**Table 2:** Partial Spearman’s correlation coefficients between plasma VLSFAs and lipids markers

VLSFAs	Fatty acids					Lipid markers			
	20:0	22:0	23:0	24:0	16:0	HDL cholesterol	LDL cholesterol	Total cholesterol	Triglycerides
20:0	1	0.7	0.69	0.61	-0.34	0.32	0.12	0.02	-0.56
22:0	0.7	1	0.75	0.93	-0.43	0.34	0.17	0.06	-0.62
23:0	0.69	0.75	1	0.73	-0.36	0.35	0.16	0.04	-0.64
24:0	0.61	0.93	0.73	1	-0.4	0.4	0.17	0.08	-0.66

AFT, Animal Fluency Test; CERAD, Consortium to Establish a Registry for Alzheimer’s Disease; CERAD-IR, CERAD immediate recall; CERAD-DR, CERAD delayed recall; DSST, Digit Symbol Substitution Test; HDL, high-density lipoprotein; LDL, low-density lipoprotein; VLSFAs, very long-chain saturated fatty acids

### Partial Spearman’s correlation analysis between plasma VLSFAs, systemic inflammatory markers, and dietary nutrients

Partial Spearman’s correlation analysis revealed that 20:0, 22:0, 23:0, and 24:0 were negatively correlated with WBC, neutrophil, and lymphocyte counts and NAR (rs = -0.13 to -0.18, -0.11 to -0.17, -0.09 to -0.11, and -0.10 to -0.16, respectively) after adjusting for age, sex, race, education, PIR, BMI, waist circumference, alcohol intake, and smoking status (Supplementary Table 7). Moreover, 24:0 was also negatively correlated with SIRI (rs = -0.1). Additionally, 22:0, 23:0, and 24:0 showed weak positive correlations with total fat, total monounsaturated fat, and total polyunsaturated fat levels in dietary nutrients (rs = 0.09–0.11, 0.08–0.12, and 0.09–0.14, respectively). However, VLSFAs had weaker and non-significant correlations with other dietary markers such as total energy intake, total cholesterol, protein, carbohydrates, fiber, and sugar (Supplementary Table 8).

### Associations between plasma VLSFAs and cognitive function after further adjusting for 16:0, HDL cholesterol, and triglycerides

**Fig. 3:**
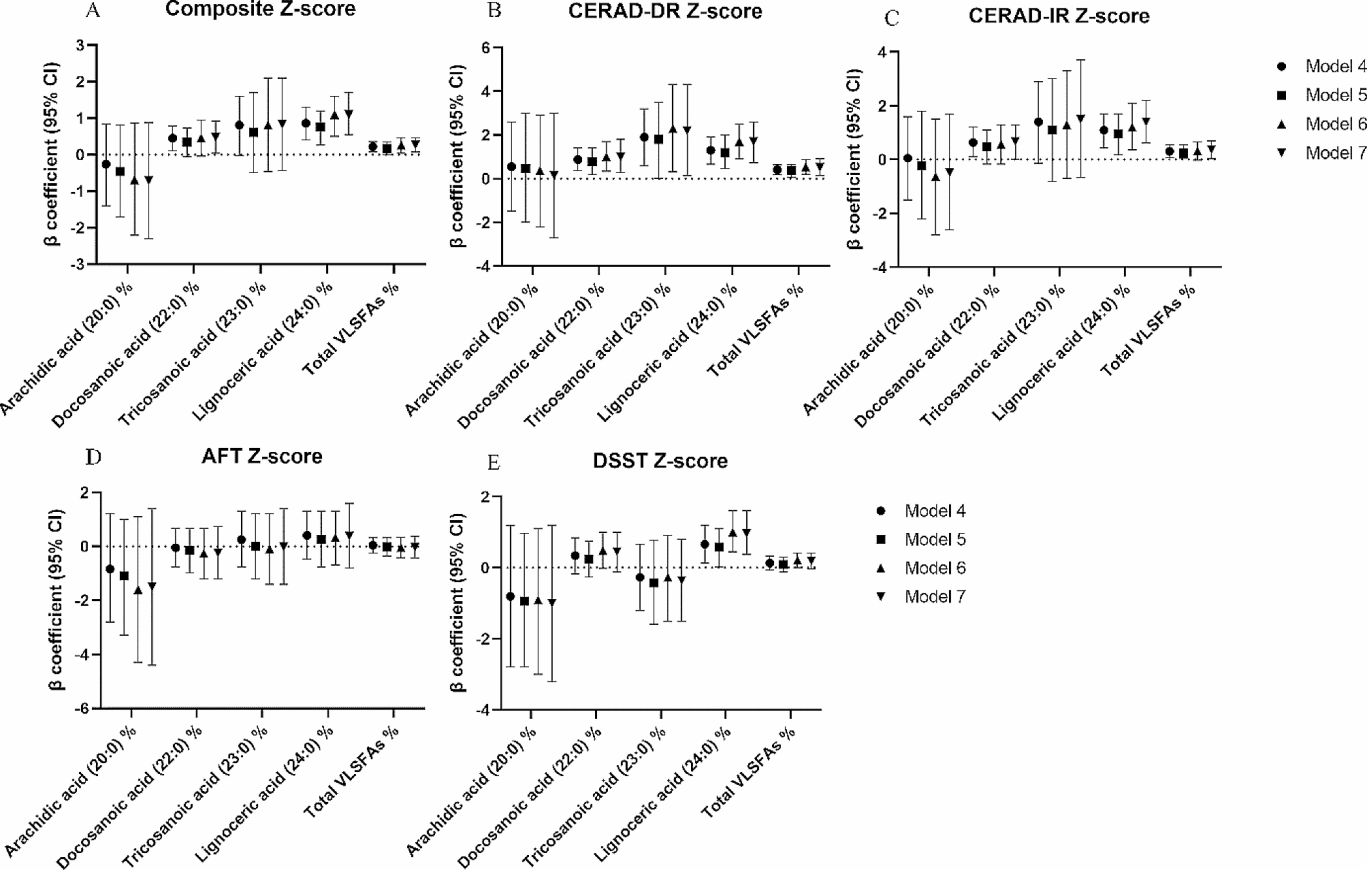
Linear regression models for associations between very long-chain saturated fatty acids (VLSFAs) and four tests of cognitive function, further adjustment for 16:0, HDL cholesterol, and triglycerides (*n* = 806). Model 4 was adjusted for 16:0 based on Model 3. Model 5 was adjusted for HDL cholesterol based on Model 3. Model 6 was adjusted for triglycerides based on Model 3. Model 7 was adjusted for 16:0, HDL cholesterol and triglycerides based on Model 3. CERAD, Consortium to Establish a Registry for Alzheimer’s Disease; CERAD-IR, CERAD immediate recall; CERAD-DR, CERAD delayed recall; HDL, high-density lipoprotein; VLSFAs, very long-chain saturated fatty acids

Figure 3. displays the associations between VLSFAs as continuous variables and cognitive performance in multivariable linear regression after further adjustment for 16:0, HDL cholesterol, and triglycerides. When we further adjusted for 16:0, it did not weaken the associations between 22:0 and 24:0 with the composite Z-score (β = 0.45, 95% CI = 0.11, 0.79 and β = 0.86, 95% CI = 0.41, 1.3, respectively) and the CEARD DR-Z score (β = 0.88, 95% CI = 0.38, 1.4 and β = 1.3, 95% CI = 0.67, 1.9, respectively). However, after adjustment for HDL cholesterol or triglycerides based on model 3, the associations between 22:0 and the composite Z-score were no longer significant (β = 0.34, 95% CI = -0.05, 0.73 and β = 0.46, 95% CI = -0.04, 0.95, respectively), but the associations between 24:0 with composite Z-score (β = 0.76, 95% CI = 0.26, 1.2 and β = 1.1, 95% CI = 0.5, 1.6, respectively) and CEARD DR-Z score (β = 1.2, 95% CI = 0.47, 2.0 and β = 1.7, 95% CI = 0.90, 2.5, respectively) were still significant. When we adjusted for all 16:0, HDL cholesterol, and triglycerides based on model 3, the associations between 22:0 and 24:0 with the composite Z-score (β = 0.48, 95% CI = 0.04, 0.92 and β = 1.1, 95% CI = 0.55, 1.7, respectively) and CEARD DR-Z score (β = 1.0, 95% CI = 0.29, 1.8 and β = 1.7, 95% CI = 0.73, 2.6, respectively) were still significant.

### Interaction analyses

We found no evidence of an interaction between each VLSFA and sex, race, education, smoking status, alcohol intake, diabetes mellitus, hypertension, stroke, or CHD in relation to associations of composite Z-scores (all *P*_value for interactions_ ≥ 0.10; Table 3).

**Table 3:** Correlations between VLSFAs and composite Z-scores in stratified analysis (*P*_value for interactions_)

Interactions	20:0	22:0	23:0	24:0
Sex	0.713	0.248	0.829	0.135
Race	0.983	0.766	0.982	0.325
Education	0.758	0.947	0.449	0.826
Smoking status	0.648	0.462	0.136	0.636
Alcohol intake	0.444	0.534	0.477	0.807
Diabetes mellitus	0.419	0.555	0.653	0.347
Hypertension	0.608	0.932	0.151	0.778
Stroke	0.18	0.252	0.995	0.36
CHD	0.747	0.579	0.35	0.699

Stratified analyses were adjusted for age, sex, race, education, PIR, BMI, waist circumference, smoking status, alcohol intake, diabetes mellitus, hypertension, stroke, CHD, and depression scores. BMI, body mass index; CHD, coronary heart disease; PIR, family income-to-poverty ratio; VLSFAs, very long-chain saturated fatty acids

## Discussion

In this large national population-based study derived from NHANES, we demonstrated that higher plasma VLSFAs (22:0 and 24:0) levels were positively associated with better global cognitive performance (composite Z-score). Similarly, higher plasma VLSFAs (22:0, 23:0, and 24:0) levels were positively associated with better delayed learning ability (CERAD-DR Z-score). In contrast, 20:0 was not significantly associated with cognitive function. Consistent with the results of several recent studies, high circulating levels of VLSFAs (22:0 and 24:0) may be beneficial in a variety of age-related diseases. In the Cardiovascular Health Study focusing on cardiovascular disease in older adults, the quintile with the highest levels of 24:0 was associated with a 16% (5–27%) reduced risk of unhealthy aging events, including cognitive decline, compared with the lowest quintile during a median follow-up of 6.4 years after adjustment for demographics, clinical conditions, and lifestyle factors [29]. In the Atherosclerosis Risk in Communities (ARIC) study of 3229 participants, a higher percentage of plasma VLSFAs in middle age was associated with less cognitive decline at 20 years [30].

The mechanisms underlying the effects of VLSFAs on cognitive function remain unclear. VLSFAs (22:0 and 24:0) are the major components of ceramides and sphingolipids and are important for myelin function [31]. Owing to differences in the length of the fatty acyl chains, different types of ceramides are thought to have different functions. Ceramides, are known for their regulatory role in apoptotic and inflammatory cell death, and appear to be associated with dementia, insulin resistance, and atherosclerosis [32, 33]. Ceramides of different chain lengths regulate apoptotic processes by differentially permeabilizing mitochondrial outer membranes [34]. Studies using animal and in *vitro* models have suggested that ceramides containing 16:0 induce apoptosis, whereas those containing 22:0 and 24:0 inhibit it [35, 36]. High levels of 22:0 and 24:0, which are associated with better cognitive function, may be related to the inhibition of apoptosis. In addition, saturated fatty acids of different chain lengths have different effects on inflammation. Specifically, 22:0 and 20:0 have a negative correlation with pro-inflammatory components and a positive correlation with the anti-inflammatory components of lipocalins. Conversely, 14:0 and 16:0 have opposite effects [18]. Increased 16:0 exposure triggers pro-inflammatory signaling pathways by binding to and activating Toll-like receptor 4 in adipocytes and macrophages, resulting in the activation of NF-κB and JNK pathways and subsequent cytokine production [37, 38]. Consistent with the results of this study, our study indicated that 22:0 and 24:0 were negatively correlated with levels of systemic inflammatory markers, albeit weakly, suggesting that the neuroprotective effect exerted by VLSFAs may be related to the suppression of inflammation. However, the mechanism behind this is unclear, as these VLSFAs are largely understudied.

Lipid metabolism and obesity also play an important role in cognitive impairment [26–28]. In our study, VLSFAs were negatively correlated with plasma 16:0, positively correlated with HDL cholesterol and negatively correlated with triglycerides. Whether 16:0 and lipid markers have an effect on the relationship between VLSFAs and cognitive performance is unclear. Previous studies have found that 16:0 potentially attenuate the associations of VLSFAs with the risk of diabetes mellitus [39]. 16:0 is a major product of fatty acid synthase and, together with triglycerides, may be markers of *de novo* lipogenesis. Thus, increased levels of VLSFAs may be a biomarker for reduced adipogenesis, leading to a reduced risk of diabetes; or VLSFAs may be more directly involved in reducing adipogenesis [13]. However, the associations of VLSFAs with heart failure were independent of 16:0 and triglyceride levels [16]. In our study, 16:0 did not affect the associations between VLSFAs and cognitive function. It suggesting that the underlying mechanisms of the associations of VLSFAs with heart failure, cognitive function and diabetes may be different. Another possible mechanism is related to fatty acid metabolism and the inhibition of lipid peroxidation. Due to the lack of very long-chain acyl-CoA synthetase in mitochondria, VLSFAs such as 22:0, 24:0, and 26:0 can only be β-oxidized in the peroxisome [40]. Plasmalogens, which are metabolites of VLSFAs, act as antioxidants, can be selectively oxidized to protect PUFAs and other membrane components from oxidation and inhibit the diffusion of lipid peroxidation, thus preventing oxidative damage to cells [41]. In addition, plasma 20:0, 22:0, 23:0, and 24:0 levels were found to be positively correlated with HDL cholesterol and negatively correlated with triglycerides. This is consistent with an observation that serum levels of both 22:0 and 24:0 were correlated with higher HDL cholesterol and lower triglyceride levels in Japanese men aged 40 years and older [42]. The mechanism behind this phenomenon may be related to PPARΔ, which activates very long-chain acyl-CoA synthetase to promote the metabolism of VLSFAs and the synthesis of plasmalogens, inhibits lipid metabolism (increasing HDL cholesterol and decreasing triglycerides) [17, 43]. HDL cholesterol, which have antioxidant, anti-inflammatory, pro-endothelial, anti-thrombotic, and immunomodulatory properties [44], have been associated with a lower risk of age-related cognitive impairment and dementia [11, 45]. However, triglycerides are associated with poorer cognitive function [46]. Although studies are not always consistent. Our study findings showed an attenuation of the associations between 22:0 and global cognitive function after adjustment for HDL cholesterol or triglycerides. This attenuation suggests that HDL cholesterol and triglycerides may mediate or confound the associations between 22:0 and global cognitive function.

Our study found that higher levels of 22:0 and 24:0 were associated with better global cognitive function, but the associations with the four cognitive domain test scores were different. 22:0 was significantly associated with delayed recall ability, but not with other cognitive domains. 24:0 was significantly associated with immediate and delayed recall, processing speed, sustained attention, and working memory. However, neither 22:0 nor 24:0 was associated with categorical verbal fluency in the executive function (Fig. 1). The physiological basis for differences in the associations of plasma VLSFAs with different domains of cognitive function remains speculative. Different brain regions regulate cognitive functions differently; for example, the medial frontal cortex and the anterior cingulate gyrus both play important roles in attention and executive control [47]. The different regions of the brain are interconnected and form a coordinated system that together regulate complex higher cognitive functions [48]. It is hypothesized that different plasma VLSFAs may have different effects on functional neural networks. In addition, there is a lack of evidence related to the quantitative analysis of the distribution of VLSFAs in different brain regions, and the mechanisms behind these observations are unclear and deserve to be confirmed by future studies. However, this study still has its important implications, and it is crucial to develop fatty acid-specific treatments based on patients with different cognitive domain impairments.

This study has several strengths. It suggests beneficial associations between 22:0 and 24:0 and global cognitive function. In addition, this study implies that it is necessary to distinguish VLSFAs from other saturated fatty acids to explore their potential health benefits. Circulating levels of VLSFAs can be altered, at least in part, by diet (e.g., by consumption of peanut butter), and the intake of such foods can be increased accordingly [49]. However, the results of the correlation between nut intake and VLSFAs were inconsistent. Although we analyzed the correlations of some dietary nutrients with VLSFAs, we did not analyze the correlations of specific foods with VLSFAs, which is important for guiding dietary interventions [50]. Further studies are needed to clarify the effects of dietary intake and endogenous metabolism on circulating levels of VLSFAs and the improvement of cognitive function. The current analysis has several limitations. The NHANES is a cross-sectional study and it does not provide evidence of causality or mechanism. Additionally, the small sample size in our study might have introduced bias and made the results less representative. It is important to conduct prospective studies with larger sample sizes in the future to gather more reliable evidence and validate the findings. Although we adjusted for sociodemographic, lifestyle, and health-related variables, it is impossible to ignore the potential influence of residual or unmeasured confounders on the results. In addition, some of the covariates included in the full model may be potential mediators, including cardiovascular risk factors and depressive symptoms. Future studies need to explore the mechanisms of action of these potential mediating variables in order to gain more insight into their explanations for our findings. Furthermore, we analyzed only plasma VLSFAs, combining other sources, such as erythrocytes, to make the results of this study more comprehensive and reliable.

In conclusion, the results of this study suggest that higher levels of 22:0 and 24:0 are associated with better global cognitive function in older adults. The associations between different types of circulating VLSFAs and cognitive function are variable. Low levels of 22:0 and 24:0 may be important biomarkers for recognizing cognitive impairment, and supplementation with specific VLSFAs (22:0 and 24:0) may be an important intervention to improve cognitive function. Our study provides valuable insights that may guide future public health recommendations on the potential benefits of VLSFAs supplementation for promoting cognitive health in older adults. Further studies are needed to clarify the underlying mechanisms by which VLSFAs affect cognitive function.

### Electronic supplementary material

Below is the link to the electronic supplementary material.

Supplementary Material 1

## Data Availability

The NHANES data are available to the public and comprehensive details regarding the collection and methodology of the survey can be found at https://www.cdc.gov/nchs/nhanes/.
