# Publisher Correction: Association between the circulating very long-chain saturated fatty acid and cognitive function in older adults: findings from the NHANES

**DOI:** 10.1186/s12889-024-18644-1

**Published:** 2024-04-22

**Authors:** Yanxin Shen, Chunxiao Wei, Yezi Taishi, Guimei Zhang, Zhan Su, Panpan Zhao, Yongchun Wang, Mingxi Li, Yingshi Ji, Li Sun

**Affiliations:** 1 Department of Neurology and Neuroscience Center, The First Hospital of Jilin University, Xinmin Street 1#, Changchun, 130021 China; 2 Cognitive Center, Department of Neurology, The First Hospital of Jilin University, Changchun, China; 3 Department of Cadre Ward, The First Hospital of Jilin University, Changchun, China; 4 Department of Pharmacology, Physiology and Cell Biology, College of Basic Medical Sciences, Jilin University, Changchun, China

BMC Public Health (2024) 24:1061

10.1186/s12889-024-18478-x

The original publication of this article contained several errors introduced during the publication process. The errors are listed in this correction article, the original publication has been rectified. The publisher apologizes to the authors & readers for the problems caused.

1. The result heading was incorrect
2. The caption of figure 1 was incomplete
3. Figure 1 was an old version

