# Supplementary material for: Association between the circulating very long-chain saturated fatty acid and cognitive function in older adults: findings from the NHANES
Source: BMC Public Health. 2024 Apr 16;24:1061. doi: 10.1186/s12889-024-18478-x (PMC11022414; doi:10.1186/s12889-024-18478-x)
Supplement: Supplementary file 1 — Supplementary Material 1 [file 12889_2024_18478_MOESM1_ESM.docx]

**Supplementary Material**

**Supplementary Table 1.** Percentage of different fatty acids in total plasma fatty acids.

| Fatty Acids | Mean ± SD % of total fatty acids |
| --- | --- |
| 20:0 | 0.21 ± 0.05 |
| 22:0 | 0.60 ± 0.16 |
| 23:0 | 0.26 ± 0.07 |
| 24:0 | 0.51 ± 0.13 |
| Total VLSFAs | 1.58 ± 0.37 |
| Total SFAs | 33.19 ± 2.21 |
| Total MUFAs | 23.1 ± 3.4 |
| Total PUFAs | 43.7 ± 4.4 |
| Total USFAs | 66.81 ± 2.21 |

MUFAs, monounsaturated fatty acids; PUFAs, polyunsaturated fatty acids; SD, standard deviation; SFAs, saturated fatty acids; USFAs, unsaturated fatty acids; VLSFAs, very long-chain saturated fatty acids.

**Supplementary Table 2.** Associations between quartiles of 22:0 % and four tests of cognitive function (n=806).

| **Quartiles of 22:0 %** | **Model 1** | | **Model 2** | | **Model 3** | |
| --- | --- | --- | --- | --- | --- | --- |
|  | **Weighted β (95% CI)** | ***P* value** | **Weighted β (95% CI)** | ***P* value** | **Weighted β (95% CI)** | ***P* value** |
| **CERAD Immediate Recall** | | | | | | |
| Q1 (<=0.49) | Reference | Reference | Reference | Reference | Reference | Reference |
| Q2 (0.49-0.60) | 0.26 (0.08, 0.45) | 0.007 ^**^ | 0.15 (-0.02, 0.32) | 0.078 | 0.17 (-0.11, 0.45) | 0.2 |
| Q3 (0.60-0.70) | 0.31 (0.07, 0.55) | 0.014 ^*^ | 0.21 (0.03, 0.38) | 0.026 ^*^ | 0.23 (-0.01, 0.46) | 0.056 |
| Q4 (>0.70) | 0.51 (0.24, 0.79) | <0.001 ^***^ | 0.24 (-0.01, 0.49) | 0.059 | 0.27 (-0.07, 0.61) | 0.10 |
| *P* trend | <0.001 ^***^ | | 0.051 | | 0.128 | |
| **CERAD Delayed Recall** | | | | | | |
| Q1 (<=0.49) | Reference | Reference | Reference | Reference | Reference | Reference |
| Q2 (0.49-0.60) | 0.15 (-0.05, 0.35) | 0.15 | 0.07 (-0.12, 0.26) | 0.4 | 0.07 (-0.20, 0.35) | 0.5 |
| Q3 (0.60-0.70) | 0.26 (0.02, 0.50) | 0.037 ^*^ | 0.20 (0.03, 0.37) | 0.023 ^*^ | 0.23 (-0.04, 0.50) | 0.082 |
| Q4 (>0.70) | 0.55 (0.31, 0.79) | <0.001 ^***^ | 0.33 (0.12, 0.53) | 0.004 ^**^ | 0.37 (0.07, 0.67) | 0.024 ^*^ |
| *P* trend | <0.001 ^***^ | | <0.001 ^***^ | | 0.007 ^**^ | |
| **Animal Fluency Test** | | | | | | |
| Q1 (<=0.49) | Reference | Reference | Reference | Reference | Reference | Reference |
| Q2 (0.49-0.60) | 0.15 (-0.03, 0.34) | 0.10 | 0.04 (-0.14, 0.22) | 0.6 | 0.05 (-0.20, 0.31) | 0.6 |
| Q3 (0.60-0.70) | 0.23 (-0.06, 0.52) | 0.11 | 0.17 (-0.08, 0.41) | 0.2 | 0.19 (-0.17, 0.55) | 0.2 |
| Q4 (>0.70) | 0.24 (-0.03, 0.50) | 0.075 | 0.04 (-0.23, 0.30) | 0.8 | 0.10 (-0.29, 0.49) | 0.5 |
| *P* trend | 0.067 | | 0.561 | | 0.900 | |
| **Digit Symbol Substitution Test** | | | | | | |
| Q1 (<=0.49) | Reference | Reference | Reference | Reference | Reference | Reference |
| Q2 (0.49-0.60) | 0.31 (0.08, 0.55) | 0.011 ^*^ | 0.14 (-0.07, 0.35) | 0.2 | 0.09 (-0.19, 0.36) | 0.5 |
| Q3 (0.60-0.70) | 0.37 (0.05, 0.69) | 0.025 ^*^ | 0.22 (-0.03, 0.46) | 0.077 | 0.16 (-0.17, 0.50) | 0.3 |
| Q4 (>0.70) | 0.59 (0.38, 0.79) | <0.001 ^***^ | 0.20 (0.04, 0.36) | 0.019 ^*^ | 0.15 (-0.09, 0.39) | 0.2 |
| *P* trend | <0.001 ^***^ | | 0.020 ^*^ | | 0.159 | |
| **Composite z** | | | | | | |
| Q1 (<=0.49) | Reference | Reference | Reference | Reference | Reference | Reference |
| Q2 (0.49-0.60) | 0.22 (0.07, 0.37) | 0.005 ^**^ | 0.10 (-0.04, 0.24) | 0.14 | 0.10 (-0.09, 0.29) | 0.3 |
| Q3 (0.60-0.70) | 0.29 (0.06, 0.53) | 0.016 ^*^ | 0.20 (0.06, 0.34) | 0.009 ^**^ | 0.20 (0.00, 0.40) | 0.049 ^*^ |
| Q4 (>0.70) | 0.47 (0.27, 0.68) | <0.001 ^***^ | 0.20 (0.04, 0.36) | 0.017 ^*^ | 0.22 (0.00, 0.44) | 0.048 ^*^ |
| *P* trend | <0.001 ^***^ | | 0.006 ^**^ | | 0.045 ^*^ | |

^*^ *P* < 0.05, ^**^ *P* < 0.01, ^***^ *P* < 0.001. BMI, body mass index; CI, confidence interval; CERAD, Consortium to Establish a Registry for Alzheimer's Disease; PIR, family income-to-poverty ratio.

Model 1, did not adjust for any confounders.

Model 2, adjusted for age, sex, race, education, and PIR.

Model 3, adjusted for age, sex, race, education, PIR, BMI, waist circumference, alcohol intake, smoking status, hypertension, diabetes mellitus, stroke and coronary heart disease, depression score.

**Supplementary Table 3.** Associations between quartiles of 24:0 % and four tests of cognitive function (n=806).

| **Quartiles of 24:0 %** | **Model 1** | | **Model 2** | | **Model 3** | |
| --- | --- | --- | --- | --- | --- | --- |
|  | **Weighted β (95% CI)** | ***P* value** | **Weighted β (95% CI)** | ***P* value** | **Weighted β (95% CI)** | ***P* value** |
| **CERAD Immediate Recall** | | | | | | |
| Q1 (<=0.41) | Reference | Reference | Reference | Reference | Reference | Reference |
| Q2 (0.41-0.50) | 0.20 (-0.02, 0.42) | 0.079 | 0.11 (-0.09,0.32) | 0.3 | 0.08 (-0.17, 0.33) | 0.5 |
| Q3 (0.50-0.60) | 0.12 (-0.11, 0.35) | 0.3 | 0.05 (-0.17, 0.26) | 0.6 | 0.02 (-0.23, 0.26) | 0.9 |
| Q4 (>0.60) | 0.60 (0.36, 0.83) | <0.001 ^***^ | 0.32 (0.09, 0.56) | 0.008 ^**^ | 0.28 (0.01, 0.55) | 0.045 ^*^ |
| *P* trend | <0.001 ^***^ | | 0.017 ^*^ | | 0.065 | |
| **CERAD Delayed Recall** | | | | | | |
| Q1 (<=0.41) | Reference | Reference | Reference | Reference | Reference | Reference |
| Q2 (0.41-0.50) | 0.14 (-0.14, 0.42) | 0.3 | 0.07 (-0.18, 0.32) | 0.6 | 0.07 (-0.24, 0.38) | 0.6 |
| Q3 (0.50-0.60) | 0.18 (-0.06, 0.42) | 0.13 | 0.14 (-0.07, 0.34) | 0.2 | 0.13 (-0.13, 0.38) | 0.3 |
| Q4 (>0.60) | 0.60 (0.40, 0.79) | <0.001 ^***^ | 0.36(0.19, 0.53) | <0.001 ^***^ | 0.36 (0.14, 0.58) | 0.006 ^**^ |
| *P* trend | <0.001 ^***^ | | <0.001 ^***^ | | 0.005 ^**^ | |
| **Animal Fluency Test** | | | | | | |
| Q1 (<=0.41) | Reference | Reference | Reference | Reference | Reference | Reference |
| Q2 (0.41-0.50) | 0.20 (-0.03, 0.43) | 0.083 | 0.13 (-0.08, 0.33) | 0.2 | 0.11 (-0.13, 0.34) | 0.3 |
| Q3 (0.50-0.60) | 0.09 (-0.21, 0.39) | 0.6 | 0.02 (-0.26, 0.30) | 0.9 | -0.01 (-0.31, 0.28) | >0.9 |
| Q4 (>0.60) | 0.43 (0.13, 0.73) | 0.006 ^**^ | 0.18 (-0.13, 0.48) | 0.2 | 0.13 (-0.22, 0.49) | 0.4 |
| *P* trend | 0.019 ^*^ | | 0.337 | | 0.545 | |
| **Digit Symbol Substitution Test** | | | | | | |
| Q1 (<=0.41) | Reference | Reference | Reference | Reference | Reference | Reference |
| Q2 (0.41-0.50) | 0.20 (-0.06, 0.46) | 0.13 | 0.08(-0.15, 0.31) | 0.5 | 0.03 (-0.26, 0.32) | 0.8 |
| Q3 (0.50-0.60) | 0.29 (0.07, 0.50) | 0.012 ^*^ | 0.16(-0.03, 0.34) | 0.087 | 0.09 (-0.15, 0.34) | 0.4 |
| Q4 (>0.60) | 0.61 (0.38, 0.84) | <0.001 ^***^ | 0.21(0.02, 0.40) | 0.035 ^*^ | 0.12(-0.12, 0.36) | 0.3 |
| *P* trend | <0.001 ^***^ | | 0.015 ^*^ | | 0.203 | |
| **Composite z** | | | | | | |
| Q1 (<=0.41) | Reference | Reference | Reference | Reference | Reference | Reference |
| Q2 (0.41-0.50) | 0.18 (-0.02, 0.39) | 0.077 | 0.10 (-0.08, 0.27) | 0.3 | 0.07 (-0.14, 0.29) | 0.5 |
| Q3 (0.50-0.60) | 0.17 (-0.01, 0.35) | 0.067 | 0.09 (-0.05, 0.23) | 0.2 | 0.06 (-0.12, 0.23） | 0.5 |
| Q4 (>0.60) | 0.56 (0.37, 0.74) | <0.001 ^***^ | 0.27 (0.12, 0.42) | 0.001 ^**^ | 0.22 (0.03, 0.41） | 0.027 ^*^ |
| *P* trend | <0.001 ^***^ | | 0.001 ^**^ | | 0.025 ^*^ | |

^*^ *P* < 0.05, ^**^ *P* < 0.01, ^***^ *P* < 0.001. BMI, body mass index; CI, confidence interval; CERAD, Consortium to Establish a Registry for Alzheimer's Disease; PIR, family income-to-poverty ratio.

Model 1, did not adjust for any confounders.

Model 2, adjusted for age, sex, race, education, and PIR.

Model 3, adjusted for age, sex, race, education, PIR, BMI, waist circumference, alcohol intake, smoking status, hypertension, diabetes mellitus, stroke and coronary heart disease, depression score.

**Supplementary Table 4.** Associations between quartiles of VLSFAs % and four tests of cognitive function (n=806).

| **Quartiles of VLSFAs**  **%** | **Model 1** | | **Model 2** | | **Model 3** | |
| --- | --- | --- | --- | --- | --- | --- |
|  | **Weighted β (95% CI)** | ***P* value** | **Weighted β (95% CI)** | ***P* value** | **Weighted β (95% CI)** | ***P* value** |
| **CERAD Immediate Recall** | | | | | | |
| Q1 (<=1.322) | Reference | Reference | Reference | Reference | Reference | Reference |
| Q2 (1.32-1.58) | 0.21 (0.00, 0.42) | 0.052 | 0.12 (-0.06, 0.31) | 0.2 | 0.14 (-0.16, 0.43) | 0.3 |
| Q3 (1.58-1.82) | 0.31 (0.06, 0.56) | 0.017 ^*^ | 0.19 (0.00, 0.38) | 0.046 ^*^ | 0.20 (-0.04, 0.44) | 0.081 |
| Q4 (>1.82) | 0.51 (0.25, 0.77) | <0.001 ^***^ | 0.25 (0.00, 0.51) | 0.053 | 0.27 (-0.09, 0.63) | 0.12 |
| *P* trend | <0.001 ^***^ | | 0.040 ^*^ | | 0.122 | |
| **CERAD Delayed Recall** | | | | | | |
| Q1 (<=1.322) | Reference | Reference | Reference | Reference | Reference | Reference |
| Q2 (1.32-1.58) | 0.11 (-0.14, 0.36) | 0.4 | 0.07 (-0.16, 0.29) | 0.5 | 0.07 (-0.26, 0.41) | 0.6 |
| Q3 (1.58-1.82) | 0.22 (-0.06, 0.51) | 0.11 | 0.16 (-0.05, 0.37) | 0.12 | 0.19 (-0.12, 0.50) | 0.2 |
| Q4 (>1.82) | 0.55 (0.32, 0.79) | <0.001 ^***^ | 0.36 (0.16, 0.55) | 0.001 ^**^ | 0.43 (0.12, 0.73) | 0.016 ^*^ |
| *P* trend | <0.001 ^***^ | | <0.001 ^***^ | | 0.004 ^**^ | |
| **Animal Fluency Test** | | | | | | |
| Q1 (<=1.322) | Reference | Reference | Reference | Reference | Reference | Reference |
| Q2 (1.32-1.58) | 0.12 (-0.10, 0.34) | 0.3 | 0.03 (-0.18, 0.24) | 0.8 | 0.03 (-0.25, 0.31) | 0.8 |
| Q3 (1.58-1.82) | 0.28 (-0.04, 0.61) | 0.082 | 0.19 (-0.05, 0.42) | 0.11 | 0.20 (-0.14, 0.53) | 0.2 |
| Q4 (>1.82) | 0.21 (-0.04, 0.46) | 0.10 | 0.02 (-0.22, 0.26) | 0.9 | 0.07 (-0.27, 0.41) | 0.6 |
| *P* trend | 0.062 | | 0.554 | | 0.968 | |
| **Digit Symbol Substitution Test** | | | | | | |
| Q1 (<=1.322) | Reference | Reference | Reference | Reference | Reference | Reference |
| Q2 (1.32-1.58) | 0.21 (-0.05, 0.47) | 0.11 | 0.08 (-0.11, 0.27) | 0.4 | 0.03 (-0.22, 0.27) | 0.8 |
| Q3 (1.58-1.82) | 0.42 (0.08, 0.75) | 0.016 | 0.23 (0.03, 0.44) | 0.029 ^*^ | 0.17 (-0.12, 0.47) | 0.2 |
| Q4 (>1.82) | 0.54 (0.31, 0.76) | <0.001 ^***^ | 0.17 (-0.01, 0.35) | 0.057 | 0.11 (-0.16, 0.38) | 0.3 |
| *P* trend | <0.001 ^***^ | | 0.023 ^*^ | | 0.205 | |
| **Composite z** | | | | | | |
| Q1 (<=1.322) | Reference | Reference | Reference | Reference | Reference | Reference |
| Q2 (1.32-1.58) | 0.16 (-0.02, 0.35) | 0.084 | 0.08 (-0.08, 0.23) | 0.3 | 0.07 (-0.15, 0.29) | 0.5 |
| Q3 (1.58-1.82) | 0.31 (0.05, 0.57) | 0.021 ^*^ | 0.19 (0.05, 0.34) | 0.011 ^*^ | 0.19 (-0.02, 0.40) | 0.064 |
| Q4 (>1.82) | 0.45 (0.26, 0.65) | <0.001 ^***^ | 0.20 (0.04, 0.36) | 0.017 ^*^ | 0.22 (-0.02, 0.46) | 0.063 |
| *P* trend | <0.001 ^***^ | | 0.003 ^**^ | | 0.041 ^*^ | |

^*^ *P* < 0.05, ^**^ *P* < 0.01, ^***^ *P* < 0.001. BMI, body mass index; CI, confidence interval; CERAD, Consortium to Establish a Registry for Alzheimer's Disease; PIR, family income-to-poverty ratio; VLSFAs, very long-chain saturated fatty acids.

Model 1, did not adjust for any confounders.

Model 2, adjusted for age, sex, race, education, and PIR.

Model 3, adjusted for age, sex, race, education, PIR, BMI, waist circumference, alcohol intake, smoking status, hypertension, diabetes mellitus, stroke and coronary heart disease, depression score.

**Supplementary Table 5.** Associations between quartiles of 20:0 % and four tests of cognitive function (n=806).

| **Quartiles of 20:0 %** | **Model 1** | | **Model 2** | | **Model 3** | |
| --- | --- | --- | --- | --- | --- | --- |
|  | **Weighted β (95% CI)** | ***P* value** | **Weighted β (95% CI)** | ***P* value** | **Weighted β (95% CI)** | ***P* value** |
| **CERAD Immediate Recall** | | | | | | |
| Q1 (<=0.18) | Reference | Reference | Reference | Reference | Reference | Reference |
| Q2 (0.18-0.21) | 0.05 (-0.14, 0.25) | 0.6 | 0.01 (-0.15, 0.16) | >0.9 | -0.03 (-0.26, 0.20) | 0.7 |
| Q3 (0.21-0.24) | 0.22 (0.06, 0.38) | 0.009 ^**^ | 0.12 (-0.03, 0.27) | 0.10 | 0.08 (-0.11, 0.27) | 0.3 |
| Q4 (>0.24) | 0.15 (-0.11, 0.42) | 0.3 | 0.05 (-0.16, 0.25) | 0.6 | 0.01 (-0.29, 0.31) | >0.9 |
| *P* trend | 0.120 | | 0.404 | | 0.714 | |
| **CERAD Delayed Recall** | | | | | | |
| Q1 (<=0.18) | Reference | Reference | Reference | Reference | Reference | Reference |
| Q2 (0.18-0.21) | 0.01 (-0.17, 0.18) | >0.9 | -0.01 (-0.20, 0.17) | 0.9 | -0.07 (-0.33, 0.19) | 0.5 |
| Q3 (0.21-0.24) | 0.13 (0.01, 0.25) | 0.033 | 0.08 (-0.07, 0.24) | 0.3 | 0.06 (-0.19, 0.31) | 0.5 |
| Q4 (>0.24) | 0.17 (-0.06, 0.39) | 0.14 | 0.12 (-0.05, 0.29) | 0.2 | 0.11 (-0.16, 0.38) | 0.3 |
| *P* trend | 0.052 | | 0.082 | | 0.218 | |
| **Animal Fluency Test** | | | | | | |
| Q1 (<=0.18) | Reference | Reference | Reference | Reference | Reference | Reference |
| Q2 (0.18-0.21) | 0.03 (-0.18, 0.24) | 0.8 | 0.01 (-0.18, 0.19) | >0.9 | -0.02 (-0.22, 0.19) | 0.8 |
| Q3 (0.21-0.24) | 0.13 (-0.09, 0.36) | 0.2 | 0.07 (-0.13, 0.27) | 0.4 | 0.07 (-0.23, 0.37) | 0.6 |
| Q4 (>0.24) | -0.11 (-0.40, 0.18) | 0.4 | -0.12 (-0.33, 0.09) | 0.3 | -0.11 (-0.44, 0.22) | 0.4 |
| *P* trend | 0.600 | | 0.367 | | 0.143 | |
| **Digit Symbol Substitution Test** | | | | | | |
| Q1 (<=0.18) | Reference | Reference | Reference | Reference | Reference | Reference |
| Q2 (0.18-0.21) | 0.11 (-0.11, 0.33) | 0.3 | 0.04 (-0.13, 0.21) | 0.6 | -0.06 (-0.27, 0.16) | 0.5 |
| Q3 (0.21-0.24) | 0.26 (-0.04, 0.55) | 0.085 | 0.10 (-0.11, 0.31) | 0.3 | 0.00 (-0.26, 0.27) | >0.9 |
| Q4 (>0.24) | 0.15 (-0.14, 0.43) | 0.3 | -0.01 (-0.22, 0.20) | >0.9 | -0.12 (-0.41, 0.17) | 0.3 |
| *P* trend | 0.200 | | 0.911 | | 0.456 | |
| **Composite z** | | | | | | |
| Q1 (<=0.18) | Reference | Reference | Reference | Reference | Reference | Reference |
| Q2 (0.18-0.21) | 0.05 (-0.11, 0.21) | 0.5 | 0.01 (-0.12, 0.14) | 0.9 | -0.04 (-0.21, 0.13) | 0.5 |
| Q3 (0.21-0.24) | 0.19 (0.04, 0.33) | 0.016 | 0.09 (-0.02, 0.20) | 0.091 | 0.06 (-0.10, 0.21) | 0.4 |
| Q4 (>0.24) | 0.09 (-0.14, 0.32) | 0.4 | 0.01 (-0.14, 0.16) | 0.9 | -0.03 (-0.24, 0.18) | 0.7 |
| *P* trend | 0.200 | | 0.580 | | 0.734 | |

^**^ *P* < 0.01. BMI, body mass index; CI, confidence interval; CERAD, Consortium to Establish a Registry for Alzheimer's Disease; PIR, family income-to-poverty ratio.

Model 1, did not adjust for any confounders.

Model 2, adjusted for age, sex, race, education, and PIR.

Model 3, adjusted for age, sex, race, education, PIR, BMI, waist circumference, alcohol intake, smoking status, hypertension, diabetes mellitus, stroke and coronary heart disease, depression score.

**Supplementary Table 6.** Associations between quartiles of 23:0 % and four tests of cognitive function (n=806).

| **Quartiles of 23:0 %** | **Model 1** | | **Model 2** | | **Model 3** | |
| --- | --- | --- | --- | --- | --- | --- |
|  | **Weighted β (95% CI)** | ***P* value** | **Weighted β (95% CI)** | ***P* value** | **Weighted β (95% CI)** | ***P* value** |
| **CERAD Immediate Recall** | | | | | | |
| Q1 (<=0.21) | Reference | Reference | Reference | Reference | Reference | Reference |
| Q2 (0.21-0.26) | 0.31 (0.11, 0.51) | 0.004 ^**^ | 0.23 (0.04, 0.43) | 0.020 ^*^ | 0.25 (0.00, 0.49) | 0.048 ^*^ |
| Q3 (0.26-0.30) | 0.40 (0.20, 0.60) | <0.001 ^***^ | 0.25 (0.05, 0.44) | 0.015 ^*^ | 0.26 (0.04, 0.49) | 0.030 ^*^ |
| Q4 (>0.30) | 0.45 (0.20, 0.70) | 0.001 ^**^ | 0.26 (0.00, 0.52) | 0.046 ^*^ | 0.27 (-0.09, 0.63) | 0.11 |
| *P* trend | 0.001 ^**^ | | 0.060 | | 0.183 | |
| **CERAD Delayed Recall** | | | | | | |
| Q1 (<=0.21) | Reference | Reference | Reference | Reference | Reference | Reference |
| Q2 (0.21-0.26) | 0.41 (0.21, 0.61) | <0.001 ^***^ | 0.34 (0.15, 0.53) | 0.002 ^**^ | 0.33 (0.06, 0.61) | 0.025 ^*^ |
| Q3 (0.26-0.30) | 0.37 (0.10, 0.63) | 0.009 ^**^ | 0.25 (0.01, 0.50) | 0.042 ^*^ | 0.28 (-0.02, 0.58) | 0.063 |
| Q4 (>0.30) | 0.52 (0.28, 0.75) | <0.001 ^***^ | 0.40 (0.18, 0.61) | 0.001 ^**^ | 0.47 (0.13, 0.81) | 0.017 ^*^ |
| *P* trend | <0.001 ^***^ | | 0.006 ^**^ | | 0.020 ^*^ | |
| **Animal Fluency Test** | | | | | | |
| Q1 (<=0.21) | Reference | Reference | Reference | Reference | Reference | Reference |
| Q2 (0.21-0.26) | 0.01 (-0.22, 0.25) | >0.9 | -0.01 (-0.21, 0.19) | >0.9 | -0.01 (-0.24, 0.21) | >0.9 |
| Q3 (0.26-0.30) | 0.20 (-0.03, 0.42) | 0.081 | 0.10 (-0.12, 0.32) | 0.4 | 0.12 (-0.18, 0.43) | 0.3 |
| Q4 (>0.30) | 0.13 (-0.16, 0.41) | 0.4 | 0.05 (-0.17, 0.26) | 0.7 | 0.12 (-0.16, 0.39) | 0.3 |
| *P* trend | 0.2 | | 0.483 | | 0.892 | |
| **Digit Symbol Substitution Test** | | | | | | |
| Q1 (<=0.21) | Reference | Reference | Reference | Reference | Reference | Reference |
| Q2 (0.21-0.26) | 0.08 (-0.18, 0.33) | 0.5 | -0.02 (-0.22, 0.18) | 0.8 | -0.11 (-0.32, 0.11) | 0.3 |
| Q3 (0.26-0.30) | 0.27 (-0.04, 0.57) | 0.085 | 0.04 (-0.18, 0.26) | 0.7 | -0.07 (-0.33, 0.20) | 0.5 |
| Q4 (>0.30) | 0.30 (0.08, 0.51) | 0.009 ^**^ | 0.01 (-0.15, 0.17) | 0.9 | -0.13 (-0.32, 0.07) | 0.2 |
| *P* trend | 0.006 ^**^ | | 0.674 | | 0.360 | |
| **Composite z** | | | | | | |
| Q1 (<=0.21) | Reference | Reference | Reference | Reference | Reference | Reference |
| Q2 (0.21-0.26) | 0.20 (0.03, 0.38) | 0.024 ^*^ | 0.13 (-0.01, 0.28) | 0.060 | 0.12 (-0.04, 0.27) | 0.12 |
| Q3 (0.26-0.30) | 0.31 (0.09, 0.52) | 0.006 ^**^ | 0.16 (-0.02, 0.34) | 0.076 | 0.15 (-0.05, 0.35) | 0.12 |
| Q4 (>0.30) | 0.35 (0.14, 0.55) | 0.002 ^**^ | 0.18 (0.02, 0.34) | 0.030 ^*^ | 0.18 (-0.03, 0.39) | 0.077 |
| *P* trend | 0.002 ^**^ | | 0.041 ^*^ | | 0.196 | |

^*^ *P* < 0.05, ^**^ *P* < 0.01, ^***^ *P* < 0.001. BMI, body mass index; CI, confidence interval; CERAD, Consortium to Establish a Registry for Alzheimer's Disease; PIR, family income-to-poverty ratio.

Model 1, did not adjust for any confounders.

Model 2, adjusted for age, sex, race, education, and PIR.

Model 3, adjusted for age, sex, race, education, PIR, BMI, waist circumference, alcohol intake, smoking status, hypertension, diabetes mellitus, stroke and coronary heart disease, depression score.

**Supplementary Table 7.** Partial Spearman’s correlation coefficients between plasma VLSFAs and inflammation markers.

| **VLSFAs** | **WBC** | **NE** | **Lym** | **NLR** | **NAR** | **SII** | **SIRI** |
| --- | --- | --- | --- | --- | --- | --- | --- |
| 20:0 | -0.14 ^***^ | -0.11 ^**^ | -0.11 ^**^ | -0.00 | -0.10 ^**^ | -0.03 | -0.05 |
| 22:0 | -0.13 ^***^ | -0.11 ^**^ | -0.09 ^*^ | -0.03 | -0.10 ^**^ | -0.01 | -0.05 |
| 23:0 | -0.18 ^***^ | -0.17 ^***^ | -0.10 ^**^ | -0.06 | -0.15 ^***^ | -0.06 | -0.10 ^**^ |
| 24:0 | -0.17 ^***^ | -0.16 ^***^ | -0.09^*^ | -0.07 | -0.16 ^***^ | -0.06 | -0.10 ^**^ |

^*^ *P* < 0.05, ^**^ *P* < 0.01, ^***^ *P* < 0.001. NAR, neutrophil-to-albumin ratio; NE, neutrophil; Lym, lymphocyte; NLR, neutrophil-to-lymphocyte ratio; SII, systemic immune-inflammation index; SIRI, systemic inflammatory response index; VLSFAs, very long-chain saturated fatty acids; WBC, white blood cell.

**Supplementary Table 8.** Partial Spearman’s correlation coefficients between plasma VLSFAs and dietary nutrients.

| **VLSFAs** | **TCHOL** | **TPFAT** | **TMFAT** | **TSFAT** | **TFAT** | **Fiber** | **Energy** | **Protein** | **Carbohydrates** | **Sugar** |
| --- | --- | --- | --- | --- | --- | --- | --- | --- | --- | --- |
| 20:0 | -0.02 | 0.02 | 0.02 | 0.01 | 0.02 | -0.02 | 0.01 | 0.01 | 0.02 | 0.04 |
| 22:0 | 0.05 | 0.14 ^***^ | 0.12 ^***^ | 0.09 ^*^ | 0.11 ^**^ | -0.01 | 0.06 | 0.05 | 0.04 | 0.03 |
| 23:0 | 0.04 | 0.09 ^**^ | 0.08 ^*^ | 0.07 ^*^ | 0.09 ^*^ | 0.03 | 0.03 | 0.04 | 0.05 | 0.01 |
| 24:0 | 0.03 | 0.13 ^***^ | 0.11 ^**^ | 0.05 | 0.09 ^**^ | 0.03 | 0.07 | 0.05 | 0.04 | 0.02 |

^*^ *P* < 0.05, ^**^ *P* < 0.01, ^***^ *P* < 0.001. TCHOL, total cholesterol; TPFAT, total polyunsaturated fat; TMFAT, total monounsaturated fat; TSFAT, total saturated fat; TFAT, total fat; VLSFAs, very long-chain saturated fatty acids.


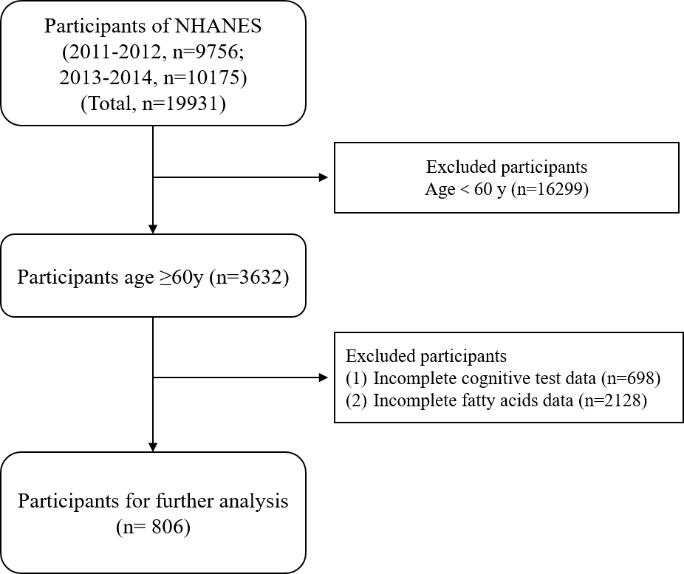


**Supplementary Figure 1.** Flow chart of study participants (2011-2012 and 2013-2014).
